# Stem cell-based therapy for hirschsprung disease, do we have the guts to treat?

**DOI:** 10.1038/s41434-021-00268-4

**Published:** 2021-06-14

**Authors:** Ali Fouad Alhawaj

**Affiliations:** 1 Department of Haematology, UCL Cancer Institute, University College London, London, WC1E 6DD United Kingdom; 2 Department of Physiology, College of Medicine, Imam Abdulrahman Bin Faisal University, Dammam, Saudi Arabia

**Keywords:** Gastrointestinal diseases, Intestinal stem cells, Intestinal stem cells, Embryonic stem cells, Embryonic stem cells

## Abstract

Hirschsprung disease (HSCR) is a congenital anomaly of the colon that results from failure of enteric nervous system formation, leading to a constricted dysfunctional segment of the colon with variable lengths, and necessitating surgical intervention. The underlying pathophysiology includes a defect in neural crest cells migration, proliferation and differentiation, which are partially explained by identified genetic and epigenetic alterations. Despite the high success rate of the curative surgeries, they are associated with significant adverse outcomes such as enterocolitis, fecal soiling, and chronic constipation. In addition, some patients suffer from extensive lethal variants of the disease, all of which justify the need for an alternative cure. During the last 5 years, there has been considerable progress in HSCR stem cell-based therapy research. However, many major issues remain unsolved. This review will provide concise background information on HSCR, outline the future approaches of stem cell-based HSCR therapy, review recent key publications, discuss technical and ethical challenges the field faces prior to clinical translation, and tackle such challenges by proposing solutions and evaluating existing approaches to progress further.

## Disease overview

### Introduction

Hirschsprung disease (HSCR), also known as colonic aganglionosis, is a congenital anomaly of the hindgut that disrupts the enteric nervous system (ENS) formation, resulting in bowel obstruction. The incidence of HSCR in the UK is 1.8 per 10,000 live births with 3.3:1 male predominance [1]. Clinically, HSCR is commonly diagnosed early after birth following delayed meconium passage. However, its presentation spans from chronic constipation that remains unnoticed until adulthood, to complete obstruction in newborns that leads to lethal enterocolitis if not promptly treated [2, 3].

The classification of HSCR is based on the extent of the aganglionic segment. Short-segment HSCR represents the majority of cases and involves the rectosigmoid area (~80% of patients). Long-segment type extends to the descending or even transverse colon (~12% of patients). Total colonic aganglionosis involves the entire colon (~8% of patients). Total intestinal aganglionosis is a rare and serious variant [4, 5]. This anatomical categorization holds critical therapeutic implications, that we will cover.

### Pathophysiology

**Fig. 1:**
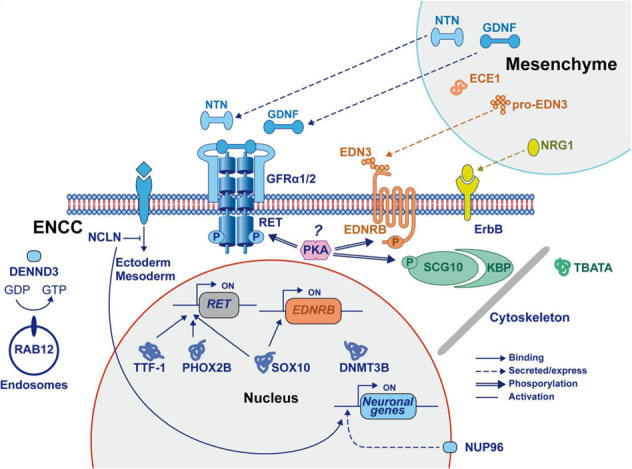
The group of identified proteins that play a role in ENS and HSCR development. The pathways are presented in the context of ENCC and gut mesenchyme interactions. The type of connection between the proteins is represented by different lines (binding, secreted/express, phosphorylation, and activation). Reproduced with permission from Gui et al. (ref. [9]).

Starting from the 4th embryonic week (EW) of development, neural crest cells (NCC) migrate from the neural tube to proliferate and differentiate along the gastrointestinal axis rostrocaudally, eventually establishing the ENS [6]. This system comprises complex networks of neuronal and glial circuitry that are responsible for the muscular and secretory functions of the gut. Upon migration, some undifferentiated NCC reside in their destination to mature into ENS lineages (termed as enteric NCC, or ENCC), while others continue the migratory wave. The mechanistic control of these dynamic processes is explained, to some extent, by signaling interactions between the NCC and the gut mesoderm. However, a full understanding of NCC gut colonization is yet to be established [7, 8]. Examples of key signaling systems that control ENS development include GDNF/RET and ET3/EDNRB pathways (Fig. 1) [9].

The GDNF/RET signaling system is composed of Glial cell-derived neurotrophic factor (GDNF) that is secreted by the gut mesoderm and interacts with the tyrosine kinase RET receptor and GDNF family receptor α1 (GFRα1) receptor that are present in the ENCC [10]. GDNF/RET signaling plays a role in chemotaxis of ENCC to migrate distally. Moreover, it promotes proliferation by acting as a mitogenic factor to ensure an adequate ENCC pool. In subsequent phases, GDNF/RET promotes neuronal differentiation [11]. ET3/EDNRB pathway is comprised of Endothelin-3 (ET3) that is secreted by the gut mesoderm to bind to EDNRB protein in the ENCC. This signaling pathway maintains ENCC progenitors in an undifferentiated state, allowing them to migrate and fully colonize the gut [12].

The primary pathology in HSCR is the failure of proper ENS formation, leading to a constricted dysfunctional segment of the bowel that causes fecal obstruction. This defect often arises from genetic defects in the pathways responsible for ENCC migration, proliferation, differentiation and survival. Nevertheless, studied cases revealed single, multiple or even no detected genetic defects, as well as the involvement of environmental factors, affirming the disease’s complex and multifactorial nature [13, 14].

The susceptibility of the distal colon in HSCR in particular is not fully understood. However, the fact that NCC migration is rostrocaudal indicates a failure of maintenance of the progenitor pool. Moreover, neuronal development in the distal gut is thought to be distinctive than other gut areas, evident by neuronal cell death in distal colon subsequent to *RET* inactivation during late development [15].

### Genetics and epigenetics

To date, around 21 genes were pinpointed in HSCR that code for proteins involved in NCC functions and ENS development pathways [9]. These mutated genes were found in around 30% of HSCR patients. The remaining unexplained risk is attributed to a combination of common and rare variants of known and undiscovered mutations [16]. HSCR occurs as an isolated trait in 70% of cases and displays variable inheritance patterns that correlate with different underlying mutations and syndromes [17]. For instance, HSCR phenotype with *RET* mutation is dominantly inherited with incomplete penetrance and is associated with MEN2A syndrome, whereas HSCR with GDNF mutation is non mendelian and non syndromic (Table 1) [18, 19].

**Table 1:** A number of HSCR genes in humans with its corresponding inheritance patterns and syndromic associations.

Gene	Inheritance	Phenotype
RET	Dominant, incomplete penetrance	Non-syndromic/MEN2A
GDNF	Non-Mendelian	Non-syndromic
NTN	Non-Mendelian	Non-syndromic
EDNRB	Recessive dominant (de novo in 80%)	Shah–Waardenburg non-syndromic
EDN3	Recessive dominant, incomplete penetrance	Shah–Waardenburg non-syndromic
PH0X2B	Dominant (de novo in 90%)	Haddad syndrome (CCHS)
SOX10	Dominant (de novo in 75%)	Shah–Waardenburg
ECE1	Dominant (de novo)	Congenital heart formation
ZFHX1B (SIP1)	Dominant (de novo)	Mowat–Wilson
KIA1279 (KBP)	Recessive	Goldberg–Shprintzen
TTF1 (TITF1)	–	Non-syndromic
NRG1	–	Non-syndromic

Reproduced with permission from Goldstein et al. [19].

*MEN2A* Multiple Endocrine Neoplasia Type 2A, *CCHS* congenital central hypoventilation syndrome.

The *RET* proto-oncogene (OMIM 164761) was found to be the major contributor to the disease phenotype, with more than 200 *RET* loss-of-function mutations linked to ~20% of the sporadic as well as up to half of the familial forms of the disease [13, 20, 21]. The reduced penetrance and phenotypic variability in *RET*-mutated HSCR could be partially explained by specific single-nucleotide-polymorphisms modifiers within the *RET* gene [22]. Moreover, the resulting phenotype could be explained by concurrent mutations, e.g., the *EDNRB* gene was shown to have an epistatic interaction with *RET* [23].

Epigenetic modifications were also investigated in the context of ENS development and HSCR pathogenesis. For instance, DNMT3B downregulation seems to contribute to the phenotypic severity through DNA methylation pattern alteration [24, 25]. Other epigenetic modifications were implicated in NCC development and function at different sites, namely histone modifications, polycomb repression, chromatin remodeling and noncoding RNA. These regulatory mechanisms are hypothesized to contribute to HSCR pathology [26].

### Current treatments

The mainstay of HSCR treatment is resection of the aganglionic segment and anastomosis of the healthy end of the colon with the anus though pull-through procedures. The general aim of surgical treatment is to relieve the obstruction while maintaining the fecal continence [27]. There are multiple surgical techniques for HSCR correction. Standard of care procedures include Duhamel, Swenson and Soave procedures. These can be performed transanally with laparoscopic assistance. Although some techniques might be superior in some patients subgroups, the treatment of choice is primarily dictated by the surgeon’s preference and expertise [28, 29].

The management of HSCR depends on the clinical presentation and the extent of aganglionosis. For uncomplicated short-segment HSCR, a single or staged corrective surgery would be undertaken in the first few months of life; otherwise, underlying complications, such as enterocolitis, needs to be treated with a possible temporary stoma creation. For total aganglionosis, the treatment comprises a decompressive ostomy and a subsequent definitive procedure performed later in life when the child grows normally, and any metabolic or nutritional derangements were resolved [30, 31].

Although the overall treatment success rate is high (mortality rate <5%) (ref. [32, 33]), HSCR is associated with significant morbidity. Studies focused on the functional aspect as a measure of outcome, demonstrate a constipation rate of 24%, fecal soiling in 21% of cases [34], and fecal incontinence in more than 50% during childhood [35], which has a devastating psychological impact. Moreover, enterocolitis was observed in 28% of cases [36]. Collectively, HSCR-associated morbidities necessitated additional surgeries in 43–48% of cases [32].

## Cell-based therapy

Progress in regenerative medicine offers cell-based solutions to the problem of ENS dysfunction, which extend beyond HSCR into other enteric neuropathies such as esophageal achalasia and gastroparesis [37]. Novel HSCR treatment strategies are of great interest as they not only avoid the adverse conventional treatment sequelae, but also aim to restore the physiological ENS development and function, at least in theory. This section discusses future cell-based therapeutic approaches of HSCR and tackles major challenges in the field.

### Approaches and cell source

The principle of the long-sought HSCR cell-based therapy is the reestablishment of a functional ENS in the aganglionic segment by harnessing the proliferative potential of ENCC. This idea, however, raises several questions that need to be addressed sequentially; what is the best source of ENCC? How could we maintain and expand ENCC in vitro prior to transplantation? Are these cells safe to use? Do we have to correct the underlying mutations in an autologous source? Would ENCC engraft into the host gut? How would we deliver those cells? Recent key publications aimed to answer some of those vital questions, with considerable advancements (Table 2). Nevertheless, before dwelling into those issues, we first must outline the therapeutic scheme that HSCR research tries to implement.

**Table 2:** Recent publications studying future HSCR cell-based therapies form multiple facets.

Study, Ref.	Platform	Question addressed
Fattahi et al. [41]	In vivo and in vitro	Generation of ESC-derived ENCC line.
		Generation of iPSC-derived ENCC line.
		The rescue of HSCR murine model.
Lai et al. [39]	In vitro	Generation of HSCR iPSC-NCC line.
		Mutation correction by CRISPR/Cas9.
		In vitro testing of ENCC migration and differentiation.
Barber et al. [40]	In vitro	Generation of iPSC-derived ENCC cell line.
Rollo et al. [60]	Ex vivo	Assessment of autologous HSCR ENCC engraftment in HSCR gut.
Cooper et al. [70]	In vivo	Transplantation of murine ENCC into an HSCR murine model.
McCann et al. [71]		
Cooper et al. [72]		Transplantation of human fetal ENCC into an immunodeficient HSCR murine model.

*ESC* Embryonic stem cell, *ENCC* enteric neural crest cell, *iPSC* induced pluripotent stem cell, *HSCR* Hirschsprung disease, *CRISPR/Cas9* clustered regularly interspaced short palindromic repeats/CRISPR-Associated Protein 9.

**Fig. 2:**
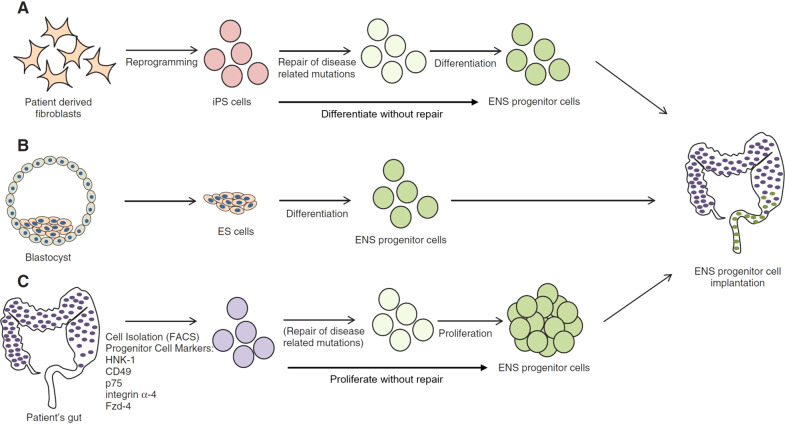
Illustration of cellular sources for ENS restoration in HSCR cell-based therapy. These approaches differ in the source of ENCC and the subsequent modifications before transplantation. Method (**A**) utilizes iPSC which could be generated from patients’ fibroblasts. Method (**B**) depends on non-autologous ES cells. Method (**C**) relies on the extraction of the progenitors from a healthy gut segment of the patient. Note that the extracted cells in method (**A**) and (**C**) might undergo gene therapy to correct the underlying mutation prior to transplantation. Reproduced with permission from Obermayr and Seitz (ref. [38]).

The envisaged therapeutic approaches could be categorized according to the source of ENCC: induced pluripotent stem cell (iPSC), embryonic stem cell (ESC), or native ENCC [38]. In the iPSC-derived ENCC implantation method (Fig. 2A), adult somatic cells, such as skin fibroblasts, would be taken from an HSCR patient and reprogramed into iPSC, associated mutations corrected ex vivo, differentiated into ENCC, and finally, modified ENCC are implanted in the aganglionic segment. One striking advantage of this method is the feasibility to obtain any somatic cell as a source and reprogram it to follow the ENS lineage. Another similar approach is to implant the iPSC-derived ENCC without attempting to correct the associated mutation. This latter issue will be covered in the *unknown mutations* section. Lai et al. generated iPSC-derived ENCC lines from HSCR patients [39] and corrected the underlying *RET* mutations by using CRISPR/Cas9. They further demonstrated the restoration of the genetically-corrected ENCC’s migration and differentiation capacity in vitro. Another group developed an enhanced differentiation protocol with a reduced time interval of 15 days [40].

With regards to ESC-derived ENCC (Fig. 2B), because the cell source would be the inner mass of a healthy human preimplantation embryo (non autologous), the treatment steps would only include differentiation and direct transplantation, bypassing the need for mutation correction. Fattahi et al. in 2016 established ESC-derived (as well as an iPSC-derived) ENS cell lineages with its specific neuronal subtypes [41], aiming to provide a drug-testing platform and a tool for HSCR cell therapy. The study went further to demonstrate the cells’ therapeutic potential by locally transplanting the derived ENCC in the colon of the HSCR mouse model *ednrb*^*s–l/s–l*^, reporting a 100% rescue rate. However, they failed to elucidate the mechanisms underlying the rescue and were subject to the confounder of significant spontaneous survival of their model [42].

The third approach is autologous ENCC transplantation (Fig. 2C). In this method, a gut biopsy is obtained from the patient’s colon to isolate the ENCC, correct the HSCR mutation, expand the cells in vitro and transplant them back into the patient. This method requires ENCC persistence postnatally, cells to be obtained by endoscopy, and isolated through specific flow cytometry markers, all of which were successfully demonstrated by Metzger et al. [43, 44]. Similar to the iPSC-driven ENCC approach, we could theoretically proceed with implantation without mutation repair. Nevertheless, in vitro proliferation protocols are likely needed due to the limited self-renewal capacity of post-natal ENCC [45].

### Safety

The safety profile of this therapy mainly relates to the cell source and mutation repair. The use of pluripotent stem cells, whether ESC or iPSC, harbors the risk of tumorigenesis [46, 47]. This risk could be minimized by the use of monoclonal antibodies to selectively ablate the undifferentiated cells pre-transplantation. Furthermore, a posttransplantation safety measure against potential tumor formation includes the use of suicide genes. This solution involves transfecting the stem cells with an apoptosis-inducing gene before transplantation, such as *CASP9*, which codes for Caspase-9 enzyme, that will be activated upon administering an inducer drug. This fail-safe measure acts as a last resort in case of unexpected severe adverse reactions when utilizing ESC or iPSC-induced ENCC [48, 49]. In addition to the tumorigenesis risk, the use of ESC-derived ENCC for allogeneic transplantation would require administering life-long immunosuppressive agents to avoid rejection and graft versus host disease [38], which might be even more harmful given the repeated bouts of enterocolitis in HSCR. This point stands in the way of attempting to recruit universal iPSC donors as well. Those concerns skew the scope of therapy, from a safety point of view at least, towards an autologous ENCC source, and iPSC to a lesser extent.

Concerning mutation repair, the potential safety risk depends on the gene therapy approach, whether utilizing gene editing or gene addition. CRISPR/Cas9 gene-editing platforms were readily adopted in biomedical research for their versatility and relative ease of use in comparison to other protein engineering techniques like Zinc finger nucleases. However, this system has variable efficiencies due to several possible outcomes of DNA repair following the DNA cut it makes [50]. Furthermore, It is prone to off-target effects, which are yet to be investigated when it was used to correct HSCR-associated mutations in iPSC in vitro [39]. These limitations could be minimized by using alternative novel CRISPR nuclease designs such as prime editing, which was developed by Anzalone et al. (ref. [51]). This system substitutes the desired DNA base by utilizing a catalytically impaired Cas9 endonuclease fused to an engineered reverse transcriptase, which could be programmed with a prime editing guide RNA. This design, unlike CRISPR/Cas9, bypasses the need for a double-stranded break and does not require a DNA template. Before attempting to apply this tool, large scale genotoxic evaluation needs to be applied, as the next-generation sequencing of PCR amplicons used by Anzalone et al. to detect off-targets is not sufficient for a genome-wide scale off-target assessment as per their evaluation [51, 52].

For gene addition, it aims to supply a functional gene product implicated in HSCR (such as *RET* gene) to ENCC via vectors. Despite the common and efficient use of viral vectors [53], they harbor the risk of insertional mutagenesis with variable degrees according to the viral vector class. For example, the treatment of X-linked severe combined immunodeficiency patients by Gammaretroviral vectors was shown to carry a significant insertional mutagenesis risk that resulted in proto-oncogene activation and the development of T-cell leukemia [54]. Further pursuits of safer vectors have led to the generation of self-inactivating (SIN) lentiviral vectors. There are two main reasons, from a safety perspective, to consider this vector class as a candidate for gene addition in HSCR. Firstly, SIN lentiviral vectors have the advantage of an approved clinical application, being the basis of KYMRIAH (Tisagenlecleucel), the first FDA-approved gene therapy for acute lymphoblastic leukemia [55]. Secondly, SIN lentiviral vectors have already been used to transfect human ENCC cells. In 2014, Natarajan et al. verified the reliability of a protocol to label ENS stem cells obtained from human colon biopsies with fluorescent reporter genes by SIN lentiviral vectors. Upon transplanting those cells in the colon of immune-deficient recipient mice, they demonstrated a transduction efficiency of ~90% that remained stable for 2 months after transplantation. The establishment of this protocol opens the venue for subsequent safety experimentation to test for the presence of insertional mutagenesis [56].

### Unknown mutations

As mentioned earlier, HSCR is a complex disease that is partially explained by isolated mutations. This fact constitutes a hurdle for gene therapy in iPSC-induced ENCC and autologous approaches. For us to undertake the commitment of repairing HSCR mutations, we would be faced, depending on the case, with either a single mutation, no identified mutations, or multiple different mutations within different genes. For single-gene mutations, gene-editing (such as by CRISPR/Cas9), or gene addition approaches (such as by viral vector delivery) are feasible. In gene addition, in particular, it is essential to consider the size of the delivery construct during vector design. For example, the estimated exome size of *RET* proto-oncogene is 31 kb that falls within 20 exons [57]. This problem of large size might be overcome by developing truncated *RET* versions, replicating the micro-dystrophin experience [58], and by optimizing the viral packaging capacity, which was attempted by Counsell et al. to increase SIN lentiviral vector maximum load [59].

For the other two groups of patients, namely who has multiple or no identified mutations, gene therapy might not be possible or feasible (given the increased off-targeting risk of multiple edits). Before coming to the conclusion that those patients are ineligible for autologous or iPSC-derived ENCC transplantation and be restricted to the ESC source, we have to establish whether gene therapy is a necessary step, i.e., whether ENCC with inherent defects can be transplanted successfully. For that, we have to keep in mind that in short-segment HSCR, which is the predominant type, ENCC were able to migrate and colonize most of the patients’ gut, rendering transplanting defective ENCC a reasonable proposition. Rollo et al. demonstrated that p75+ ENCC isolated from HSCR patients are capable of engrafting in an autologous aganglionic segment ex vivo [60]. However, whether this proliferative capacity of defective cells is specific to certain mutations or generalized is a matter of future investigation.

In another broader front, unbiased GWAS studies are vital for novel gene discovery, especially when coupled with next-generation whole-genome or whole-exome sequencing technologies [16, 61]. In HSCR, multiple GWAS studies were performed by utilizing microarray genotyping to identify associated variants [62–64], and more recently, WES and WGS were used in trio design to identify novel genes and variants [9, 65, 66].

Studying the genetic landscape of HSCR could provide a better understanding of the clinical significance of detected variants. For instance, certain genotypes might be linked to a higher incidence of postoperative enterocolitis, which could be considered in the treatment algorithm and follow-up [21]. Moreover, The role of genetic testing and prenatal diagnosis could be expanded, as it is currently hindered by the variable penetrance of pathogenic mutations, and the incomplete understanding of many seemingly benign variants [67]. Challenges include: (1) the recruitment of a sample size that achieves a satisfactory statistical power when examining rare variants in a GWAS design, (2) meeting the high cost of next-generation sequencing in such a sample, and (3) determining whether nongenetic “environmental” factors contribute to the disease etiology, which is still to be elucidated.

### Engraftment

The concept of ENCC engraftment into a host gut embeds several components that have been studied over time, namely anatomical engraftment, functional integration, the extent of coverage, and interaction with the host gut.

Successful transplantation of ENCC in wild-type mice was demonstrated [68, 69]. To progress further, studies resorted to HSCR mouse models to study aspects of engraftment and explore the treatment potential even further. In 2016, Cooper et al. investigated in vivo transplantation of murine ENCC into HSCR *ednrb*^*tm1Ywa*^ mice that lack functional endothelin receptor type-B [70]. Immunohistochemistry showed formation and colonization of neurons and glia with branching ENS-like network over an area of 4.3 ± 3.1 mm^2^ in 56/62 animals (90.3%). Functional integration was demonstrated by calcium fluorescence imaging, where electrical stimulation was applied to endogenous neurons and Ca^2+^ response recorded from the transplanted cells. In terms of safety, a 2-year follow-up showed no tissue spread or tumor formation. This result is in accordance with the findings of McCann et al. who transplanted murine ENCC into a neuronal nitric oxide synthase-deficient mouse model (*nNOS*^*−/−*^) that recapitulates human the HSCR phenotype of gut motility disturbance [71]. This study showed a rescue of the phenotype-associated gut motility function, evident by improved contractile properties and decreased total intestinal transit time. Furthermore, the study reports an exciting finding of extensive trans-colonic engraftment of ENCC despite utilizing identical protocols in previous work, owing to the use of confocal microscopy imaging to examine whole colonic preparations instead of the flawed approach of stereoscopic live imaging that lead to underreporting of coverage in other studies.

To explore the potential of human ENCC as a cellular source, Cooper et al. transplanted human fetal human ENCC donated from human fetal colons into an immunodeficient HSCR mouse model [72]. Engraftment was reported in only 50% of the mice, with axonal projections of differentiated neurons extending over 1.2 ± 0.6 mm. The authors attributed this limited engraftment to the inherent variability of human samples and decreased robustness of human cells to proliferate in vitro and form neurosphere-like bodies in comparison to the mouse ENCC. It is reasonable to assume that this limitation was also influenced by the difference in the species’ niches.

Challenges in this domain include upscaling the extent of human ENCC coverage to produce a clinically significant effect in future therapies in a proportionally longer human gut [73]. Although Rollo et al. transplanted human autologous ENCC into its corresponding aganglionic segment in HSCR patients, the fact that the recipient gut segments were cut into 1–2 mm^3^ fragments makes it difficult to assess for the maximum amount of the gut that would be reconstituted by donor cells [60]. Moreover, little is known about the potential aberrant interaction between the transplanted cells and the new host gut microenvironment and whether that will lead to the transplant establishing a network that functionally competes with the host ENS or properly engrafts and integrates [74].

The anatomical and functional development of ENS was tracked in both murine and human contexts [75, 76]. Utilized methods include immunohistochemistry for neuron subtypes detection, calcium imaging for examining the coordinated neuronal activity, and transcriptional analysis (such as for genes encoding for ion channels) to study the transcriptional alterations that coincide with the emergence of electrical activity. Since the final aim is to establish functional ENS that resembles the physiological one, the engraftment of ENCC could be further investigated by comparing it to the physiological ENS development at the molecular and functional levels, as mere engraftment of ENCC might not reflect an organized ENS. Functional testing will be mentioned at the end of this section.

We could hypothesis that murine ENS formation posttransplantation would follow a similar pattern to that of normal development, measured by methods mentioned above (immunohistochemistry, calcium imaging, and transcriptional analysis), which is a similar experimental approach that was performed by McCann et al. on human embryonic tissue [76]. One suggested setup is to compare ENS development between transplanted ENCC in an aganglionic mouse model with ENCC from a healthy control at different time points. Fluorescence-Activated Cell Sorting could be utilized to isolate donor cells at fixed intervals (e.g., pretransplant, [D0]; 1 week, 2 weeks, 3 weeks, 4 weeks, and 3 months posttransplant) and isolate ENCC from a reporter mouse at, for example, E12.5, E14.5, E18.5, D0. The cells would be taken from an equivalent environment, such as the distal colon. By using single-cell RNA sequencing, we could ensure examining the cells at a more precise level, as they would contain a heterogeneous population. Important considerations include the choice of ENCC marker, the location, and the intervals at which the tissue is obtained and assessed. Also, as ENCC expansion protocols are being refined, it would be interesting to compare different ENCC sources in parallel in terms of engraftment efficiency and success rate.

As previously mentioned, Cooper et al. examined the engraftment of murine ENCC by immunohistochemistry and calcium fluorescence imaging [70]. One addition to this design could be to perform murine ENCC transplantation (whether healthy, defective, or corrected) in an aganglionic mouse model alongside a control arm without transplantation. Cooper et al. argued that using HSCR models, such as *ednrb*^*s–l/s–l*^, would prove to be hard in such studies because of its early death, limiting the time of assessment and follow-up [70]. While McCann et al. used *nNOS*^*−/−*^ mouse model that is characterized by an increased lifespan to better assess the transplant outcome [71], Stamp et al. proposed a surgical manipulation to *ednrb*^*s–l/s–l*^ by creating a stoma to alleviate the luminal obstruction and prolong its survival from ~1 to 5 weeks [77]. The latter approach would retain the HSCR-associated neuropathy more accurately yet prolong survival to better evaluate the outcomes of interventions.

Moreover, as a safety indicator, we could concomitantly generate a differentially expressed gene list from transcriptional analysis of murine colonic samples posttransplantation against ones from the control mouse model, such as by RNA sequencing, and search for the enrichment of oncogenic transcriptional patterns. Such exploration would require running the differentially expressed gene list in a functional bioinformatic analysis tool such as over-representation analysis or gene-set enrichment analysis along with published transcriptional datasets of murine colorectal cancer [78].

With regard to motility, propulsion motility assessment techniques, such as spatiotemporal mapping, could be applied to a whole colon organ bath posttransplantation [79]. This experiment could examine the functional behavior of the transplanted ENS and look for any changes in the motility pattern that may affect the propagation of luminal content. While these experiments are necessary to understand how to manipulate and study the transplantation process, there is a need to transfer the studies to intact human recipient colons, which faces an ethical barrier. This will be discussed in the following section.

### Ethical considerations

Among the cellular sources of ENCC, the use of ESC is highly controversial. In fact, the opinions formulated on whether to accept destroying an early human embryo to cure disease and alleviate suffering are deeply rooted in moral beliefs, resulting in discrepant policies worldwide that make a consensus unlikely. As a result, iPSC present as a more morally acceptable alternative [80].

The second ethical consideration is the assessment of the risk-to-benefit ratio before proceeding with clinical trials [81]. In the HSCR scenario, almost all patients will undergo at least one type of procedure, which, as stated earlier, has an overall excellent survival rate of ≥95% (ref. [32, 33]). This current outcome triggers the question of whether it is justified to conduct a first-in-human cell therapy trial against a necessary effective curative surgery. To tackle this issue, we could design a trial where the new cell-based therapy will not compete with the standard of care, instead, integrating it as an adjuvant to the conventional treatment, therefore, minimizing the risk of depriving the patient of necessary treatment. This trial design could recruit HSCR patients with extensive variants of the disease who would otherwise undergo an early ostomy (where the distal aganglionic colon is left) and a definitive surgery to anastomose the healthy segment with the distal gut later in life [31]. In those patients, ENCC could be transplanted in the distal unresected aganglionic part before undergoing the definitive second surgery, hoping for ENS restoration and functional integration with the anastomosed segment without delaying or replacing the standard treatment.

### Delivery methods

Delivery of therapeutic cells, regardless of the source, has two aspects to consider. First, whether there is an optimal histological location of administration that will yield a better therapeutic outcome, and second, which technical method would be preferred to deliver the therapy in humans. Studies in animal models directly injected cell suspensions or neurospheres-like bodies via laparotomy. Preliminary evidence points towards a preference of gut muscular [41] and subserosal injections [70] over the peritoneal route [82], with no established preferred target gut layer.

Cheng et al. proposed utilizing endoscopy as a safe and reliable delivery method by delivering ENCC to healthy and HSCR mice, demonstrating successful engraftment in 9/12 mice with no complications [83]. According to the authors, the failure of engraftment in the remaining three mice is attributed to the technical difficulty of targeting the thin gut of the mouse model. The potential of endoscopic delivery could be further studied by testing on large animal models where endoscopic ultrasonography could be applied to examine layer-specific delivery [83, 84].

## Conclusion

This review demonstrates how our understanding of gut development and HSCR pathogenesis amalgamate to pursue a future cell-based cure. After casting an overview of HSCR in the context of ENS pathways and genomic disturbances, the outcome of current treatment options was demonstrated. Lastly, different cell-based therapy approaches were addressed as an alternative cure, along with several questions that remain unanswered in the field.

Earlier work generated ENCC cell lines and rescued gut motility in animal models. However, research at the frontline is yet to fully establish the necessary tools, protocols, and the treatment potential and applicability of each of the discussed approaches. The next major leap lies within advancing the proposed therapies to human experimentation. This upscaling will be possible after improving our understanding of the ENS and HSCR development and studying the integration of transplanted ENS progenitors from different sources and monitoring its consequences. By enhancing current treatment approaches and eliminating therapeutic safety concerns, this therapy will teach us not only how to cure HSCR, but to even approach other conditions as to how to extract, correct, proliferate, differentiate, deliver, integrate, and treat with stem cells.
